# Utility of a virtual small group cognitive behaviour program for autistic children during the pandemic: evidence from a community-based implementation study

**DOI:** 10.1186/s12913-024-11033-9

**Published:** 2024-05-30

**Authors:** Vivian Lee, Nisha Vashi, Flora Roudbarani, Paula Tablon Modica, Ava Pouyandeh, Teresa Sellitto, Alaa Ibrahim, Stephanie H. Ameis, Alex Elkader, Kylie M. Gray, Connor M. Kerns, Meng-Chuan Lai, Johanna Lake, Kendra Thomson, Jonathan A. Weiss

**Affiliations:** 1 Department of Psychology, Carleton University, 214E Social Science Research Building, Ottawa, ON K1S 5B6 Canada; 2 Department of Psychology, York University, 230 BSB, 4700 Keele St, Toronto, ON M3J 1P3 Canada; 3 Centre for Addiction and Mental Health, 1001 Queen Street West, Toronto, ON M6J 1H4 Canada; 4 Department of Psychiatry, Temerty Faculty of Medicine, University of Toronto, 250 College Street, 8th Floor, Toronto, ON M5T 1R8 Canada; 5 Kinark Child and Family Services, 7271 Warden Ave, Markham, ON L3R 5X5 Canada; 6 Centre for Education Development, Appraisal, and Research, University of Warwick, Coventry, CV4 7AL UK; 7 Department of Psychology, University of British Columbia, 2136 West Mall, Vancouver, BC V6T 1Z4 Canada; 8 Department of Applied Disabilities Studies, Brock University, 1812 Sir Issac Brock Way, St. Catherines, ON L2S 3A1 Canada; 9 Azrieli Adult Neurodevelopmental Centre at the Centre for Addiction and Mental Health, 1451 Queen Street West, Toronto, ON M63 1A1 Canada

**Keywords:** Autism, Children, Virutal CBT, Emotional regulation, Social skills, Community services

## Abstract

**Background:**

Autistic children often experience socioemotional difficulties relating to emotion regulation and mental health problems. Supports for autistic children involve the use of adapted interventions that target emotion regulation and social skills, alongside mental health symptoms. The Secret Agent Society Small Group (SAS: SG), an adapted cognitive behavioural program, has demonstrated efficacy through lab-delivered randomized control trials. However, research is still needed on its effectiveness when delivered by publicly funded, community-based autism providers under real-world ecologically valid conditions, especially within the context of a pandemic. The COVID-19 pandemic has disrupted access to community-based supports and services for autistic children, and programs have adapted their services to online platforms. However, questions remain about the feasibility and clinical utility of evidence-based interventions and services delivered virtually in community-based settings.

**Methods:**

The 9-week SAS: SG program was delivered virtually by seven community-based autism service providers during 2020–2021. The program included the use of computer-based games, role-playing tasks, and home missions. Caregivers completed surveys at three timepoints: pre-, post-intervention, and after a 3-month follow-up session. Surveys assessed caregivers’ perception of the program’s acceptability and level of satisfaction, as well as their child’s social and emotional regulation skills and related mental health challenges.

**Results:**

A total of 77 caregivers (94% gender identity females; *Mean* = 42.1 years, *SD* = 6.5 years) and their children (79% gender identity males; *Mean* = 9.9 years, *SD* = 1.3 years) completed the SAS: SG program. Caregivers agreed that the program was acceptable (95%) and were highly satisfied (90%). Caregivers reported significant reduction in their child’s emotion reactivity from pre- to post-intervention (-1.78 (95% CI, -3.20 to -0.29), *p* = 0.01, *d =* 0.36), that continued to decrease after the 3-month booster session (-1.75 (95% CI, -3.34 to -0.16), *p* = 0.02, *d =* 0.33). Similarly, improvements in anxiety symptoms were observed (3.05 (95% CI, 0.72 to 5.36), *p* = 0.006, *d* = 0.39).

**Conclusions:**

As online delivery of interventions for autistic children remains popular past the pandemic, our findings shed light on future considerations for community-based services, including therapists and agency leaders, on how best to tailor and optimally deliver virtually based programming.

**Trial registration:**

This study has been registered with ISRCTN Registry (ISRCTN98068608) on 15/09/2023. The study was retroactively registered.

**Supplementary Information:**

The online version contains supplementary material available at 10.1186/s12913-024-11033-9.

## Introduction

Autistic children often experience difficulties with emotion regulation and social communication skills, which can interfere with their functioning and have a negative impact on their quality of life and well-being. Difficulties in emotion regulation (i.e., challenges in monitoring, evaluating, and expressing one’s own emotions [16]) are considered transdiagnostic symptoms [1, 37] in that they are implicated in the development of many different mental health problems, including anxiety, depression, eating disorders, and substance use [18]. Emotion regulation is also often relational in nature [16], and in autistic children, challenges with emotion regulation have been correlated with greater social communication difficulties [25]. Pandemic-related policies (e.g., closure of schools and community-based services, lockdowns, etc.) meant to limit the spread of COVID-19 likely exacerbated the emotion regulation problems, as well as social and mental health difficulties experienced by many autistic children [23, 24, 27, 28, 36, 39].

For verbally able autistic children, variations of adapted cognitive behaviour therapy (CBT) programs have been used to improve emotion regulation skills and social skills, alongside mental health problems. For instance, work from Wood and colleagues [44] demonstrated the effectiveness of the Behavioral Interventions for Anxiety in Children with Autism (BIACA), an intervention delivered in modular format that allows social skills to be targeted alongside coping skills for anxiety. The study found that BIACA was more effective in increasing social communication skills when compared to traditional CBT programs that focused on anxiety reduction alone. Similarly, White et al. [40, 42] demonstrated the feasibility and clinical utility of the Multimodal Anxiety and Social Skills Intervention (MASSI), an adapted CBT program that considers the interconnectedness of anxiety and social communicative challenges in autistic children. Beyond solely treating anxiety, group-based CBT programs have also been successfully adapted to target emotion regulation and social skills [7, 22, 32, 35]. A randomized control trial of a one-on-one CBT program, the Secret Agent Society: Operation Regulation (SAS: OR) [3], showed improvements in emotion regulation and adaptive skills, and reductions in externalizing symptoms and overall psychiatric symptom severity [38].

Pandemic disruptions have accelerated the need for programs that leverage existing online platforms to deliver therapeutic interventions, including using synchronous (real-time) and asynchronous (recorded) sessions, homework assignments, and peer support [2, 17]. Even before the pandemic, emerging evidence supported the effectiveness of online-based programs. For example, Beaumont and colleagues [6] conducted a pilot randomized control trial of an online version of the Secret Agent Society Small Group (SAS: SG) program [4, 33] for autistic children within a university-setting and found improvements in parent-reported social skills and problem behaviours compared to a control group. Lee and colleagues [21] conducted a mixed-methods evaluation of an online SAS: OR program during the first wave of the pandemic and demonstrated improvements in emotion regulation, social skills, and reductions in children’s externalizing behaviours after participation in the intervention. Other programs that target social skills and anxiety were also quickly adapted for online delivery, and preliminary results demonstrated general improvements in target behaviours (PEERS - Lee et al., 2023 [20]; Facing Your Fears - McMorris et al., in prep). Although the results of these pilot programs are promising, there is still a need to explore considerations for delivering virtual programming, particularly in community-based settings where autistic children receive most of their supports.

In Canada, community-based agencies are often publicly funded and provide the bulk of services for autistic children (e.g., behavioural interventions and supports, family workshops, parent respite, core clinical services, etc.). During the pandemic, many of these agencies continued to provide adapted virtual supports (e.g., phone consultations, online programming, etc.) for families. Group programs that are delivered by community agencies have been particularly impacted by the pandemic, as lockdowns and social distancing measures limited the availability of services [21, 29]. There is some research suggesting that in-person community agencies were among the first to close and one of the last to re-open following pandemic restrictions in Canada [45], relative to hospital or school-based programs (Data from the Canadian Institute for Health Information, see https://www.cihi.ca/en for more information).

Online delivery of programs by community services can be beneficial and help address logistical barriers that many families face [11, 27]. Online platforms may enhance intervention adherence and accessibility, as participants can access services from their homes, reducing barriers related to transportation, resources, and time [6, 11, 23]. Such interventions can also be tailored to the unique needs and preferences of families, including the ability to access services outside geographical location or service boundaries (e.g., catchment area) and to participate in sessions without leaving their home [2, 6, 23]. Yet changes to evidence-based interventions for online delivery, especially within the context of a pandemic, require careful considerations of feasibility and intervention clinical utility.

The current study reports on the feasibility and clinical utility of an adapted virtual socioemotional intervention (SAS: SG) delivered during the pandemic by seven community agencies in Ontario, Canada. Using an effectiveness-implementation hybrid design [10], which takes a dual focus by testing the effects of a clinical intervention on relevant participant outcomes while gathering information on implementation. For this study we tested the effectiveness of participation on child socioemotional and clinical outcomes (i.e., parent-reported changes in emotion regulation and social skills, and symptoms of anxiety and depression) post-intervention and after a 3-month follow-up session. At the same time, we gathered information on the feasibility of the program’s delivery by assessing the level of intervention acceptability reported by families, session attendance, therapist fidelity, and parent ratings of intervention acceptability and satisfaction.

## Method

### Participants

Families were eligible to participate in the intervention if: (a) their child was between 8 and 12 years of age; (b) the child had a confirmed autism diagnosis from a regulated healthcare professional; (c) caregivers informally reported child difficulties with emotion regulation and social functioning, and/or were waiting for supports to address emotion regulation and social skills; and (d) a caregiver was able to participate in the program. Families were excluded if the child had (a) an intellectual disability; (b) a diagnosis of acute psychosis or conduct disorder; or (c) any behaviours that made online group participation a safety concern (e.g., self-harm behaviours, etc.).

A total of 87 families, across 7 agencies, participated in the study. Ten did not complete the intervention (see results section for more information about non-completing families). Of the 77 primary caregivers (94% mothers; *Mean*_*age*_ = 42.5 years, *SD*_*age*_ = 5.7 years) who completed the program, 67 completed the optional 3-month follow-up booster session. Caregivers identified as primarily White (72%), South/West/East Asian (12%), multiethnic (5%), Latin American/Hispanic (5%), and Black (2%). Children (79% identified their gender as males; *Mean*_*age*_ = 9.9 years, *SD*_*age*_ = 1.3 years) identified as White (66%), multiethnic (17%), South/West/East Asian (9%), Black (2%) and Latin American/Hispanic (2%). Additional participant characteristics are presented in Table 1.

**Table 1:** Caregiver and Child Demographics

Variables	Mean (SD) or %	Range
Age (years)		
Child	9.9 (1.3)	8–13
Caregiver	42.1 (6.5)	28–58
Gender Identity (Female)		
Child	22%	
Caregiver	94%	
Autism Characteristics		
SRS-2 T-Score	71.3(8.9)	53–90
SCI T-Score	70.23 (8.9)	54–90
RRB T-Score	71.76 (9.6)	46–90
Ethnicity (identified as ethnically diverse)		
Child	36%	
Caregiver	28%	
Caregiver marital status (married)	77%	
Caregiver graduated from college	55%	
Family income		
< $49,999	13%	
$50,000 - $99,999	20.8%	
$100,000 - $149,000	20.8%	
$150,000 - $200,000+	22.1%	
Prefer not to disclose	15.6%	

*Note* SRS-2 T-Score = Social Responsiveness Scale, 2nd Edition, Total T-Score. SCI = Social Communication and Interaction T-Score, RRB = Restricted Interests and Repetitive Behaviours T-Score. Ethnicity diverse means participants who identified as non-white. Family Income is in Canadian Dollars

### Procedure

The study was approved by the research ethics board at the researchers’ institution, an academic hospital, and by the research review committee at two community-based agencies. The project was supported by a community-partner participatory framework [19], and was co-designed by a team of researchers, as well as with community agency leadership and frontline staff (e.g., therapists, child, and youth workers, etc.). Prior to implementation, researchers met with agencies to discuss agency-specific recruitment strategies and protocols, and how best to incorporate the intervention into existing programming without interfering with overall service deliverables.

Seven community-based autism service providers across Southern Ontario participated in the implementation of the SAS: SG project between October 2020 and December 2021. Prior to the delivery of each group, 21 therapists participated in a standardized four-day online training in August 2020 facilitated by the SAS: SG development team. Please see the Appendix to review therapist demographics including their level of education and discipline of practice.

Families were screened and recruited by each agency. Agencies followed their usual screening and enrollment protocol, as outlined by their own agency guidelines and policies, for offering services to children and their families on their client list. In publicly funded service providing agencies, children only require an autism diagnosis to get access to services and supports, and do not have to meet clinical cut-offs to enroll in interventions targeting emotion regulation and social skills. Therapists will use clinical judgement to determine which programs would best match the child’s and/or family needs. In some agencies, caregivers can self-refer their child if they feel that the focus of a program might be a good fit for their child. Some participants were recruited internally from agency waitlists, and some agencies recruited participants using social media posts or emailing past clients. All families were screened according to the inclusion and exclusion criteria. Once a family was deemed a good fit for the program by the SAS: SG therapist team (e.g., ready to receive intervention, available for group sessions, family goals align with program targets, etc.), researchers contacted the participants to review the research consent and provide details for participation in the study. Caregivers were then sent the pre-intervention child and family measures to be completed online. Caregivers completed post-intervention child and family measures (see below for program description and delivery schedule), and again after the 3-month follow-up booster session.

### Intervention

The *Secret Agent Society: Small Group Program* (SAS: SG; Social Science Translated) [3–5] is a spy-themed manualized cognitive behavioural program focused on helping school-age children with identified emotion regulation and social skill difficulties. All caregiver and child sessions were delivered virtually through Zoom or Microsoft Teams. The program included separate caregiver and child sessions facilitated by therapists from each agency, as well as between-session practice activities and inclusive classroom tip sheets for each child’s schoolteachers. Agencies had the option to deliver the parent and child group sessions at the same time, or on different days, but the modules were synchronized to ensure that the parent session reviewed concepts covered in the child group sessions. Child sessions targeted social communication skills, working on teams, problem solving, developing and maintaining friendships, recognizing emotions in oneself and others, coping with feelings of anger and anxiety, and expressing emotions in helpful ways (for more specific information about the intervention, see https://www.secretagentsociety.com/). The child sessions were either provided as a weekly 9-session (90 min per session) or an 18-session (45 min per session) format. In the current study, out of 77 children, 65 (84.4%) received the 9-session format and 12 (15.6%) received the 18-session format. In the 9-session format, 92.3% attended at least 8 sessions or more, and in the 18-session format, 83% attended at least 16 sessions or more. The sessions were facilitated by either one or two trained facilitators, with a group of 3–4 or 4–6 children.

Caregiver sessions reviewed key components from the child sessions and teach caregivers how to support generalization of skills at home and beyond. Caregiver sessions were delivered in three different formats, and agencies could choose the schedule that worked best for them. The formats included (1) 9 weekly sessions of 45 min per week; (2) 18 weekly sessions of 30 min per week; or (3) three 2-hour sessions every 3 weeks. All agencies offered a 2-hour parent information session prior to beginning the program. In the current study, 56 (72.7%) caregivers received the 9 sessions module, 12 (15.6%) caregivers received the 18 sessions module, and 9 (11.7%) received the three 2-hour sessions. In the 9-session format, 96.4% attended at least 8 sessions or more; in the 18-session format, 83% attended at least 16 sessions or more; and in the 3-session format, 55.5% attended at least 2 sessions or more.

### Measures

#### Implementation measures

*Attendance.* Therapists tracked attendance for the weekly sessions and the 3-month booster session.

*Fidelity*. Therapists tracked their adherence to the SAS: SG protocol using a weekly session checklist. The checklists were collected after completion of the program, and fidelity was calculated as the percentage of completed tasks across all sessions.

*Implementation Acceptability Scale (IAS)* [23]. The IAS is a 7-item lab-developed measure to assess intervention acceptability at the end of the 9-week sessions, based on Sekhon and colleagues’ theoretical framework of acceptability [31]. Caregivers were asked to describe their experience receiving the intervention using a five-point Likert scale (1 = “*strongly disagree*” to 5 = “*strongly agree*”), with higher scores reflecting greater treatment acceptability. Caregivers rated various dimensions, including affective attitude, burden, ethicality, intervention coherence, opportunity costs, perceived effectiveness, and self-efficacy. We evaluated acceptability based on the percentage of respondents that at least indicated “*agreed*” or higher (e.g., 3 or higher on the scale) for each question.

*Program Satisfaction Questionnaire (PSQ)* [3]. Caregivers completed the PSQ, which assessed their views on the appropriateness and effectiveness of the program. Open-ended questions asked caregivers to comment on changes in their child’s skills or behaviour, confidence in supporting their child, enjoyment of the program, and satisfaction with the therapists. Caregivers were also asked to describe their satisfaction with different components of the program on a five-point Likert scale (0 = “*not at all satisfied*” to 5 = “*very satisfied*”). The program components included: format, session dates and times, number of sessions and session length, and overall program satisfaction.

#### Child outcome measures

*The Social Responsiveness Scale 2nd Edition (SRS-2)* [9]. The SRS-2 is a 65-item caregiver-report measure used to capture school-aged (4–18-year-olds) children’s social functioning and autism-related characteristics. Caregivers are asked to respond on a 4-point Likert Scale (0 = “*Not True*” to 3 = “*Almost Always True*”) to statements related to their child’s social functioning including in areas of social awareness, social cognition, social communication, social motivation, and the presence of restricted interests and repetitive behaviours. The SRS-2 has good external reliability ( 0.90), with strong internal consistency [9]. Additionally, this measure has high predictive validity (0.92) and construct validity [9]. It is one of the most widely used measures of children’s actual social performance and it can be expected to show moderate to large changes in the context of a successful clinical intervention [43]. In the current study, the Total T-score, Social Communication and Interaction (SCI) scale T-score and the Restricted Interests and Repetitive Behavior (RRB) scale T-score were used.

The *Emotion Dysregulation Inventory (EDI)* [26]. The EDI is a caregiver-report measure developed to assess the severity of autistic children’s struggles with negative mood and reactivity via two subscales, Dysphoria (6 items; anhedonia, sadness, nervousness) and Reactivity (7 items; explosive outbursts, difficulty calming, rapid escalation, intense/extreme/inappropriate emotionality). Items are rated on a 5-point Likert scale (0 *= “not at all”* to 4 *= “very severe”*). The items were summed for each subscale and converted to T*-*Scores. For both subscales, higher scores indicated greater dysregulation. The EDI shows strong validity and reliability for assessing Reactivity and Dysphoria in autistic children [26]. Internal consistency for Dysphoria and Reactivity within the current sample pre-intervention were very good: α = 0.88 and α = 0.89, respectively.

*Child and Adolescent Symptoms Inventory-5* (*CASI-5)* [13]. The CASI-5 is a caregiver-report measure that gathers information about the symptoms of Diagnostic and Statistical Manual and Mental Disorders- 5th Edition (DSM-5) defined disorders in children and adolescents between the ages of 5 to 18 years. The 173-item inventory is organized into modules where each consists of a list of symptom statements for 14 of the most commonly defined DSM-5 disorders. Caregivers are asked to rate whether their child displays any of the symptoms on a 4-point scale ranging from “*never*” to “*very often*”. The tool shows strong validity and reliability in caregivers of autistic children, including overlap with interview measures of mental health disorders [12]. This measure has been found to have very good internal consistency for assessments of anxiety (α = 0.85-0.88) [14] and depression (α = 0.83) [15] in parent-reports. In the current study, we used the total symptom severity T-Score for separation anxiety, social anxiety, generalized anxiety disorder, and major depression disorder, with higher scores indicating greater level of presenting symptoms.

### Data analyses

All analyses were conducted using SPSS version 28. Implementation acceptability and feasibility were explored using descriptive statistics, while changes in child outcome measures were analyzed using paired t-tests and repeated-measures ANOVAs, with a Greenhouse-Geisser correction to account for violations of sphericity. Post hoc analyses of ANOVA outcomes used Bonferroni corrections.

## Results

### Non-completer participant profiles

A preliminary evaluation of the demographic profiles and key baseline characteristics of families included those who did not complete the program are outlined in Table 2 (*n* = 10). Families listed various reasons for being unable to continue with the program including scheduling issues with the group sessions (*n* = 2), the program required too much time commitment (*n* = 2), lack of interest in the theme (*n* = 1), virtual format not a good fit for their child (*n* = 3), and urgent family obligations (*n* = 2). These caregivers attended on average 2.43 sessions (*Range* = 1–3) and children attended 1.67 sessions (*Range* = 0–4). There were no significant differences in age or gender distributions between non-completer and completer caregivers. Independent sample t-tests showed a significantly higher SRS-2 RRB T-score for non-completer children (*M* = 78.3, *SD* = 9.60) compared to completer children (*M* = 78.30, *SD* = 9.03, *F* (1,86) = 4.14, *p* = 0.04). There were no other significant differences in pre-intervention child outcome measures or child demographics.

**Table 2:** Child and caregiver demographics and key outcome variable means and ranges at pre-program for *non-completer* families of the SAS: SG program

Variables	Mean (SD) or %	Range
Age (years)		
Child	10.3 (1.2)	
Caregiver	38.8 (11.9)	
Gender Identity (Female)		
Child	30%	
Caregiver	100%	
SRS-2 T-Scores		
Total	76.4 (9.8)	61.0–90.0
SCI	75.2 (10.6)	58.0–90.0
RRB	78.3 (9.0)	62.0–90.0
EDI T-Scores		
Reactivity	51.2(11.3)	45.9–54.9
Dysphoria	50.8 (10.0)	36.4–65.9
CASI T-Scores		
Separation Anxiety	61.8 (11.)	50.0–78.0
Social Anxiety	62.0 (9.0)	50.0–76.0
GAD	70.4 (9.2)	50.0–78.0
Depression	65.8 (12.9)	50.0–78.0

### Implementation results

As shown in Table 3, almost all treatment completers attended their weekly parent and child group sessions (attended 93.8% of sessions).

**Table 3:** Caregiver and child weekly attendance by agency program schedule

Session Version	*n*	> 80% Attendance (%)	Range
Child Sessions			
9 session	65	86%	3–9 sessions
18 session	12	100%	15–18 sessions
Caregiver Sessions			
3 2-hour session	7	100%	2–3 sessions
10 session	53	83%	5–10 sessions
18 session	12	100%	16–18 sessions

On the self-reported checklists, therapists indicated above 80% fidelity for both caregiver (*M* = 93.5%, *Range* = 88–99%) and child (*M* = 86.6%, *Range* = 78.9–6.9%) weekly sessions. A review of the fidelity checklists suggested that therapists were unable to complete some parts because of technology issues that prevented the completion of certain activities (e.g., online board game, virtual missions with the group, poor internet connections preventing participation, etc.), ran out of time to do an activity during the session (which resulted in assigning the task as homework), and/or unexpected disruptions (e.g., child abruptly disengages from the group, home-based interference, etc.). In terms of post-program acceptability, 75% of caregivers agreed or strongly agreed that they felt positively about the program, 95% agreed or strongly agreed that it aligned with their values, 87% agreed that they understood how it worked, 77% agreed that they did not have to give up resources or opportunities to participate in the program, and 77% agreed that they felt confident in the skills they had learned. Lower ratings of acceptability related to acceptable amount of effort to participate (only 61% agreed) and feeling that it was effective in achieving its goals (only 62% agreed). A qualitative analysis of caregiver feedback (*n* = 20) indicated that the virtual format required parents to spend more time monitoring their child’s group sessions in order to manage their behaviours, and to help them stay engaged. Some caregivers (*n* = 10) hoped that participation would lead to new emotion regulation or social skills but instead were somewhat disappointed when the program only reinforced their child’s current skill set. On the post-intervention PSQ, 70% of the caregivers reported feeling “*moderately*” to “*very*” confident in their ability to support their child’s future social and emotional development following completion of the program, and 83% reported that the program was “*moderately*” to “*very*” enjoyable for their child. Caregivers reported being “*moderately*” to “*very*” satisfied (90%) with their SAS: SG group facilitator, and overall, 71% of caregivers reported being “*moderately*” to “*very*” satisfied with the entire program.

### Child outcomes

The pre-intervention SRS-2 Total T-score ranged from 54 to 90 (*M* = 71.33, *SD* = 8.91). For the SCI scale, the pre-intervention t-score ranged from 54 to 90 (*M* = 70.52, *SD* = 9.07) and the RRB scale T-score ranged from 52 to 90 (*M* = 72.03, *SD* = 9.28). In our sample, 91% of children met clinical level of concern on the SRS-2 Total T-Score. As shown in Table 4, there was a significant difference between SRS-2 Total T-Scores, SCI, and RRB T-scores across pre-, post-, and the 3-month time points. Scores consistently decreased over time, which demonstrated improvements from pre- to post-intervention (Mean Difference = -3.14 (95% CI, -7.86 to -4.60, *p* = 0.001, *d* = 0.55)), and from post-intervention to the 3-month booster session (Mean Difference = -2.76 (95% CI, -5.03 to -0.49, *p* = 0.001, *d* = 0.52)) on the SRS-2 Total T-score.

On the emotion dysregulation measure (EDI), there were significant differences between time points on the EDI Reactivity and Dysphoria T-scores. The pre-intervention EDI Reactivity T-scores ranged from 30.1 to 66.7 (*M* = 50.13, *SD* = 7.07) and the EDI Dysphoria T-score ranged from 36.4 to 70.3 (*M* = 48.01, *SD* = 8.84), with 52% and 25% of the children in our sample meeting clinical cut-offs for emotion regulation difficulties across the two scales, respectively [8]. EDI Reactivity scores decreased from pre-intervention to post-intervention (-1.78 (95% CI, -3.2 to -0.29), *p* = 0.01, *d =* 0.36), and continued to decrease from post-intervention to after the 3-month booster session (-1.75 (95% CI, -3.34 to -0.16), *p* = 0.02, *d =* 0.33). While EDI Dysphoria scores decreased from pre-intervention to post-intervention, this change was not significant. EDI Dysphoria scores continued to decrease after the 3-month booster, with scores being significantly lower than the pre-intervention scores (-3.13 (95% CI, -5.02 to -1.23), *p* = 0.001, *d =* 0.51) but not the post-intervention scores (-1.67 (95% CI, -3.40 to 0.56, *p* = 0.061).

Scores on the CASI-5 indicated that pre-intervention, 38% of the children met clinical range of concerns for separation anxiety (*T*-score range = 50.0–78.0), 49% for social anxiety (*T*-score range = 50.0–76.0), 83% for general anxiety disorder (*T*-score range = 50.0–78.0), and 43% for depression (*T*-score range = 50.0–78.0). There were significant changes across time points with respect to symptoms related to both generalized anxiety disorder (GAD) and major depression, but not for separation anxiety and social anxiety. GAD symptom severity scores improved from pre-intervention to post-intervention (3.05 (95% CI, 0.72 to 5.36), *p* = 0.006, *d* = 0.39), and then remained stable from post-intervention to the 3-month booster session (0.80 (95% CI (-3.38 to 1.77), *p* = 1.00). For depression, there appeared to be no statistically significant change from pre- to post-intervention (2.29 (95% CI, -0.56 to 5.16), *p* = 0.16), but there was a significant improvement from pre-intervention to the 3-month booster session (3.61 (95% CI (0.84 to 6.38), *p* = 0.006, *d =* 0.39) (See Table 4).

**Table 4:** Pre-, post-, and 3-month caregiver-reported measures of child outcomes

Variables	*n*	Pre-Intervention Mean (SD)	Post-Intervention Mean (SD)	3-month Booster Mean (SD)	F Statistic
SRS-2 T-score					
Total	67	71.49 (9.0)	68.34 (9.3)	65.25 (9.9)**	41.68 **
SCI	67	70.52 (9.0)	67.59 (9.3)	64.58 (9.9)**	37.24 **
RRB	67	72.02 (9.3)	69.01 (9.5)	66.25 (10.3)**	22.06 **
EDI T-score					
Reactivity	66	50.2 (6.8)	48.4 (6.8)	46.6 (7.2)**	15.02**
Dysphoria	66	48.0 (8.6)	46.6 (8.0)	44.9 (7.6)**	9.14**
CASI-5 T-score					
Separation Anxiety	67	59.8 (10.3)	58.6 (9.3)	58.5 (9.5)	n.s.
Social Anxiety	52	60.6 (9.3)	59.9 (10.5)	58.3 (9.0)	n.s.
GAD	67	67.5 (9.3)	64.4 (9.5)	63.6 (9.7)**	5.29*
Depression	66	61.0 (11.3)	58.7 (10.6)	57.4 (9.8)**	7.63**

* = *p* < 0.05, ** = *p* < 0.001, n.s. = *p* > 0.05

## Discussion

The present study used an effectiveness-implementation hybrid design to evaluate the effects of an adapted virtual cognitive behaviour program, SAS: SG, on autistic children’s socioemotional outcomes while collecting information on community-based implementation. The SAS: SG program is publicly available, but existing research has largely focused on outcomes from lab- or university-based evaluations, and prior to the COVID-19 pandemic, it was delivered primarily in an in-person format. Findings from the current study suggest that the program was implemented successfully with high therapist-reported fidelity across seven community autism-focused service agencies and provides support for more rigorous research into the efficacy of group-based online programs for autistic children in the community. Results from our study suggest that families completed most of their weekly parent and child group sessions, with similar attendance rates compared to in-person adapted group programs [44] and other online versions of the program [23]. The program also saw a rather low attrition rate (∼ 10%) which may have reflected the strengths of an online formatting that decreased the usual barriers to participation including the cost of travel (e.g., time and financial costs).

In terms of feasibility, caregivers rated most aspects of intervention acceptability as high and described feeling positively about the program, that it aligned with their values, and that they understood how the program worked. However, it should be noted that a substantial group of caregivers did not agree that the program demanded a reasonable amount of effort from them (39%) or that it was effective in achieving its goals (38%). A review of text-based comments from caregivers indicated that the amount of time that was asked of them was sometimes overwhelming, including having to monitor their child’s participation, learn new concepts, support their child’s learning of skills, and facilitate assigned home activities on a weekly basis. For some caregivers, this led to hours of work above and beyond their own participation in the parent groups. These themes are consistent with previous findings related to delivering caregiver-involved online programs during the pandemic [17, 23, 41]. In terms of goal achievement, some caregivers were underwhelmed by the usefulness of the skills taught in the program. A qualitative review of text responses from caregivers indicated that that some hoped that their child could have learned new emotion regulation and social skills rather than practice skills they had already mastered. Some caregivers felt that the online format did not provide enough opportunities for their children to practice and apply the social skills being taught in the program, thus not achieving their original goals.

Considering caregiver feedback about the program, it is important for virtually delivered programs to consider the demands placed on caregivers that build upon existing stressors in their life [21]. During the pandemic, this reflected the additional demands of managing online support for their children, the evolving virtual school requirements, on top of their own work and household responsibilities, COVID-related illness, or other stressors. There is literature highlighting how this added burden is often placed upon primary caregivers, usually mothers. There is an urgent need to acknowledge these considerations around equity of supports for caregivers during the pandemic and beyond [30]. A review of non-completing families suggests that children who had higher levels of RRBs had a more difficult time engaging in online sessions. This is consistent with previous work [23] suggesting that program delivery with an online format may not be well suited to all caregivers and autistic children, especially those with behaviours that interfere with sitting and attending (e.g., compulsive behaviours, self-injurious behaviours, etc.).

Although there are benefits with delivering a program online (e.g., limiting the cost of travel, enabling further research for community service providers, etc.), some families may require different supports to make participating online more accessible to them. This might include adaptations like shorter sessions, more frequent assessment of motivation and engagement, greater use of specialized interests, and adaptations that focus on individualized care. For example, Mootz et al. [27] described modifications made to optimize participation for a single group pilot SAS: SG program delivered via telehealth in Australia during the pandemic. They described similar needs to develop procedures to support families including troubleshooting technology throughout delivery, shortening sessions, and tasking caregivers with supervision of child sessions (e.g., giving out end-of-session rewards, specifying consequences for non-engagement, etc.). Yet, despite online adaptations some children and their families may still find in person programming more beneficial and better suited to their needs. Future research by the current team includes a direct comparison of in person versus online version of the SAS: SG program in community-based services.

It should be noted that the current study took place at the beginning of the pandemic when rolling lockdowns were prevalent and there were few competing activities for families that required travel (e.g., other appointments, recreational activities, etc.). During this time, some caregivers were actively seeking access to any programs for their children, which may have contributed to the rather high engagement with the virtual program (e.g., lower than usual attrition rate). In addition, reduced demands associated with online delivery of the program (i.e., less travel time) may have contributed to increased feasibility and satisfaction with the program.

Successful implementation of this program may be the result of a community-partnered participatory framework that allowed each agency flexibility in recruitment and delivery [19]. Agencies managed their own scheduling (e.g., number of weeks, days, and times, etc.), and were supported by the research team throughout the project (e.g., troubleshooting technology issues, etc.). Therapist-reported fidelity suggested that adherence was high (87% or higher), although we could not independently verify their session fidelity as sessions were not recorded. Therapists did face some challenges completing parts of the modules, especially during child sessions, due to technology issues, running out of time, and unexpected disruptions. These are important factors to consider from an implementation perspective for future hybrid service delivery. Anecdotally, feedback from therapist teams suggests that those who spent more time troubleshooting and preparing for technological issues were able to react and respond better when issues arose during program delivery. Some therapists noted that caregiver involvement was necessary to deescalate emotionally tense situations with their children, which, as noted, often increased the demands placed upon caregivers. Therapists mentioned that specialized interests were incorporated into group sessions as necessary, and overall, most worked hard (e.g., provided visual aids, used animations, and used an abundance of reinforcers and/or tokens, etc.) to engage the children in the group sessions. Therapists should consider individual child and family needs, and screening participants for suitability for online-based group programs should consider access to technology and a family’s ability to support their child’s participation in the program [13, 23].

In terms of clinical utility of the program, caregivers reported improvements in child emotion regulation and social communication skills from pre- to post-intervention, and these gains were sustained after the 3-month booster sessions. Caregivers also reported that their children showed statistically significant improvements in social interactions and communication behaviours, and emotion reactivity at each time point. Emotion dysphoria, however, revealed a different pattern, as scores did not show a statistically significant improvement until after the 3-month booster. These findings are consistent with the main tenets of the SAS: SG program which aims to teach children deescalating techniques to prevent emotionally reactive behaviours in the face of intense or socially frustrating situations, and these skills may take additional time to solidify.

Consistent with previous work [17], no changes were found in separation and social anxiety at post-intervention and following the 3-month booster session. Given that many children were at home during the early waves of the pandemic, they may have had fewer opportunities to socialize, or be separated from caregivers. Caregivers did report improvements in children’s generalized anxiety symptoms, even after the 3-month booster session. Similar to the emotional dysphoria findings, caregivers reported steady improvements in symptoms of depression over time, but the scores reached statistically significant levels of improvement only after the 3-month booster session. These findings may suggest that the program may indirectly benefit dysphoric or behaviours resulting in negative moods and, with practice, symptoms improve over time, even though it does not specifically target them.

Interpretation of the results should be mindful of a few study limitations. The study was a single-arm implementation trial which makes our results particularly suspectable to placebo effects, and results were interpreted without a control group or blinded independent clinical assessments. Data from the study were based mainly on caregiver reports which may differ from therapist and child perspectives. Fidelity ratings were self-reported by therapists and could not be independently verified by recordings, and future implementation trials would benefit from independent coding of recorded sessions for reliability. Interpretation of the findings should consider the variability in symptom severity of our sample, especially since not all children met clinical levels of concern pre-intervention, on emotion dysregulation (52% for reactivity and 25% for dysphoria), and mental health symptoms (e.g., only symptoms of generalized anxiety were above clinical threshold pre-intervention, with social anxiety and depression symptoms being moderately elevated). Although our results showed post-intervention improvements across these domains with small to moderate effect sizes, they may not reflect a clinically meaningful change as expected for most interventions. Clinically meaningful improvements in symptoms related to generalized anxiety, however, were observed, suggesting that the socioemotional support program may have some indirect impact on improving some aspects of mental health. Finally, there seems to be particular risks in overinterpreting improvements post-intervention that may simply be due to family acclimations to pandemics stressors. Although, the SAS: SG program was delivered between September 2020 to September 2021, and started at least 4 months after the initial shut-downs due to the pandemic, which suggests that families had some time to adjust.

Despite these limitations, our findings suggest that an evidence-based intervention targeting emotion regulation and social skills in autistic children is feasible and can be delivered by community-based service providers with success. To our knowledge, this is the first study to demonstrate the effectiveness of an adapted, virtual group-based program focused on socioemotional skills, delivered in the community for autistic children during the early waves of the pandemic. Results highlight the need for ongoing support for autistic children, especially given the unpredictable circumstances imposed by the past and future pandemic resulting in a global loss of supports (e.g., therapy, social skills groups, academic and recreational programming). Our findings encourage community-partnerships with publicly funded agencies and contribute to the emerging efforts to narrow the gap from research to practice in implementing evidence-based programs in community settings. Ultimately, training in evidence-based programs by community providers can increase access to helpful ways of supporting emotion regulation and social challenges for autistic children.

### Electronic supplementary material

Below is the link to the electronic supplementary material.

Supplementary Material 1

## Data Availability

The datasets generated and/or analysed during the current study are not publicly available due university-imposed restrictions around data sharing agreements but are available from the corresponding author on reasonable request.
