# Supplementary material for: Utility of a virtual small group cognitive behaviour program for autistic children during the pandemic: evidence from a community-based implementation study
Source: BMC Health Serv Res. 2024 May 30;24:685. doi: 10.1186/s12913-024-11033-9 (PMC11140895; doi:10.1186/s12913-024-11033-9)
Supplement: Supplementary file 1 — Supplementary Material 1 [file 12913_2024_11033_MOESM1_ESM.docx]

**Table 1**

***Therapist Demographics (n = 21)***

|  | *Mean* (*SD)* or % |
| --- | --- |
| Age | 33.57 (9.72) |
| Gender |  |
| Female | 85.7 |
| Male | 14.3 |
| Education |  |
| Some college/university | 9.5 |
| Bachelor’s Degree | 38.1 |
| Master’s Degree | 47.6 |
| Doctoral Degree | 4.8 |
| Employment status |  |
| Full time | 95.2 |
| Part time | 4.8 |
| Years of Practice |  |
| 0-2 years | 9.5 |
| 3-5 years | 19.0 |
| 6-9 years | 38.1 |
| 10-14 years | 19.0 |
| 15-19 years | 9.5 |
| 20+ years | 4.8 |
| Therapeutic Orientation |  |
| Behavioral | 71.4 |
| Cognitive Behavioral | 19.0 |
| Dynamic/Analytic | 4.8 |
| Eclectic | 4.8 |
| Professional Group |  |
| Child and Youth Worker | 19.0 |
| Social Worker | 14.3 |
| Clinical Psychologist | 4.8 |
| ABA Therapist | 9.5 |
| Behavior Analyst | 19.0 |
| Behavior Therapist | 23.8 |
| N/A or none | 9.6 |
